# Molecular genetic and geochemical assays reveal severe contamination of drinking water reservoirs at the ancient Maya city of Tikal

**DOI:** 10.1038/s41598-020-67044-z

**Published:** 2020-06-25

**Authors:** David L. Lentz, Trinity L. Hamilton, Nicholas P. Dunning, Vernon L. Scarborough, Todd P. Luxton, Anne Vonderheide, Eric J. Tepe, Cory J. Perfetta, James Brunemann, Liwy Grazioso, Fred Valdez, Kenneth B. Tankersley, Alison A. Weiss

**Affiliations:** 1 Department of Biological Sciences, University of Cincinnati, Cincinnati, OH 45221 USA; 2 Department of Plant and Microbial Biology and the BioTechnology Institute, University of Minnesota, St. Paul, MN 55108 USA; 3 Department of Geography, University of Cincinnati, Cincinnati, OH 45221 USA; 4 Department of Anthropology, University of Cincinnati, Cincinnati, OH 45221 USA; 5 National Risk Management Research Laboratory, US Environmental Protection Agency, Cincinnati, OH 45224 USA; 6 Department of Chemistry, University of Cincinnati, Cincinnati, OH 45221 USA; 7 Museo Miraflores, 7 Calle 21-55, Guatemala City, Guatemala; 8 Department of Anthropology, University of Texas, Austin, TX 78212 USA; 9 Department of Molecular Genetics, Biochemistry and Microbiology, University of Cincinnati, Cincinnati, OH 45267 USA

**Keywords:** Environmental impact, Socioeconomic scenarios, Sustainability, Plant sciences, Environmental social sciences

## Abstract

Understanding civilizations of the past and how they emerge and eventually falter is a primary research focus of archaeological investigations because these provocative data sets offer critical insights into long-term human behavior patterns, especially in regard to land use practices and sustainable environmental interactions. The ancient Maya serve as an intriguing example of this research focus, yet the details of their spectacular emergence in a tropical forest environment followed by their eventual demise have remained enigmatic. Tikal, one of the foremost of the ancient Maya cities, plays a central role in this discussion because of its sharp population decline followed by abandonment during the late 9^th^ century CE. Our results, based on geochemical and molecular genetic assays on sediments from four of the main reservoirs, reveal that two of the largest reservoirs at Tikal, essential for the survival of the city during the dry seasons, were contaminated with high levels of mercury, phosphate and cyanobacteria known to produce deadly toxins. Our observations demonstrate severe pollution problems at a time when episodes of climatic aridity were prevalent. This combination of catastrophic events clearly threatened the sustainability of the city and likely contributed to its abandonment.

## Introduction

The ancient Maya abandoned the major center of Tikal in the mid-ninth century CE and although scholars have studied this site intensively for the past 60 years, exactly how and why the city met its ill-fated ending have remained unanswered questions. Our investigations, combining novel aDNA and soil geochemistry assays, however, shed significant new light on the abandonment of this once-powerful political, ceremonial and commercial hub. Recent explanations for the downfall of Tikal have centered on population expansion^1,2^ coupled with extensive landscape degradation^3,4^ and a period of multidecadal droughts from 820 to 870 CE^5–11^. Extended episodes of climatic aridity made the city especially vulnerable because it lacked access to permanent bodies of water such as lakes or rivers, and the groundwater table, approximately 200 m below the surface, was inaccessible using existing technology^12^. Inhabitants relied on reservoirs that ringed the city and filled during the rainy season to provide water during the dry season^13–15^. To enhance this water collection process, numerous large paved plazas in the site core of Tikal, e.g., the Great Plaza, the Plaza of the Seven Temples and the West Plaza^14–16^ were canted to drain water into the reservoirs, particularly the Temple and Palace Reservoirs, during the rainy season for storage^14^.

**Figure 1:**
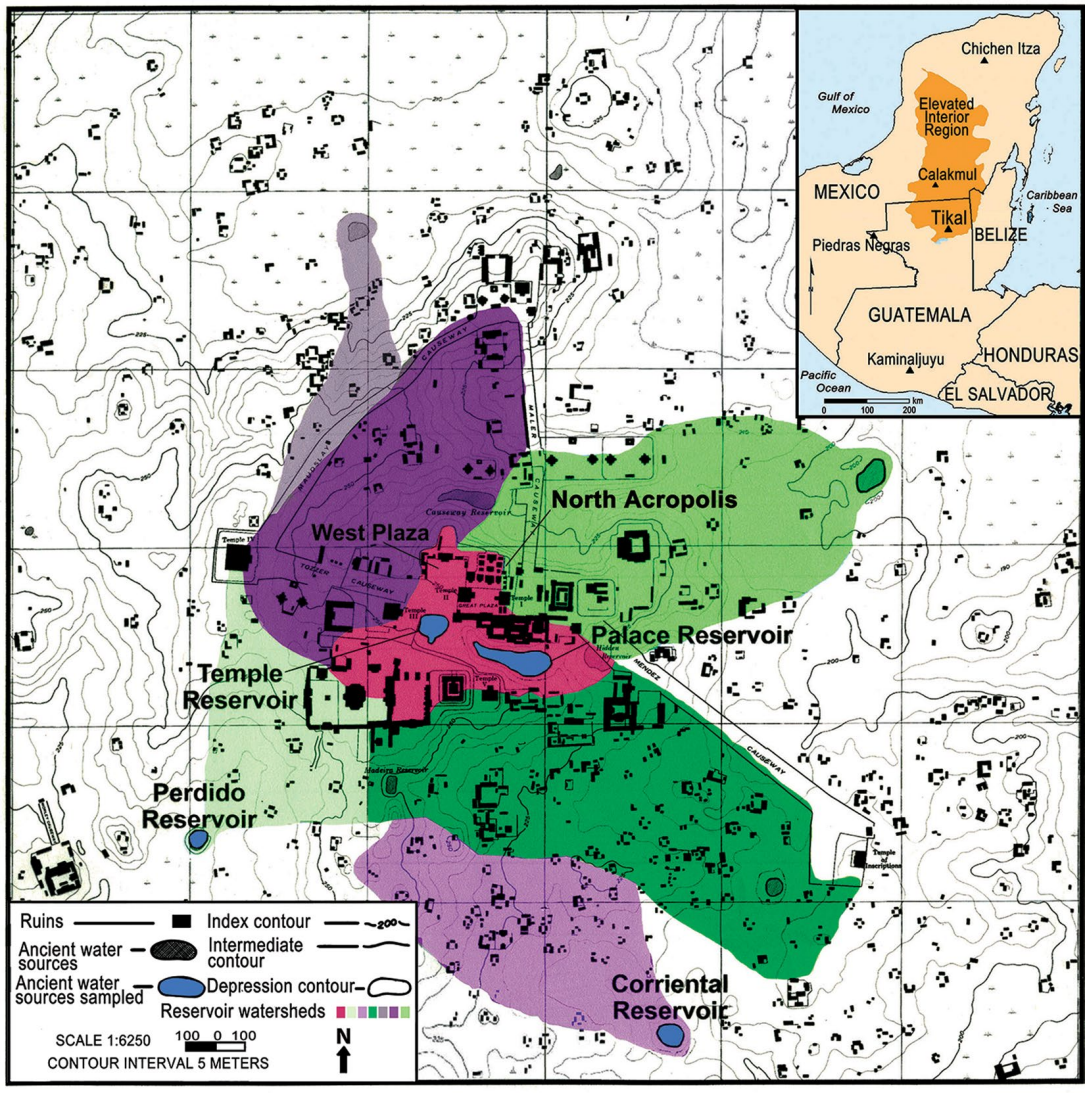
Watershed map of the site core of Tikal. The red area in the middle of the map represents the portion of the site core that drains into the Temple and Palace Reservoirs. Redrawn by Cory Perfetta and David Lentz using Photoshop CS6, Vers. 13.0.1 × 64 (https://www.adobe.com/products/photoshop/pricing-info.html). Modified from a source map (Fig. 2) in Scarborough and Gallopin^15. ^Reprinted with permission from AAAS.

In this study, we seek to bring about a greater understanding of the reservoir system at Tikal and how it played a crucial role in sustaining the inhabitants. In particular, we were interested in how the reservoirs were used and how they were maintained as sources of potable drinking water and water for irrigation. Towards this end, we collected sediment samples during excavations in 2009 and 2010 from deep strata from within four Tikal reservoirs, viz., Palace, Temple, Perdido and Corriental (Fig. 1). The first two reservoirs were located in the site core of the city while the latter two were more peripheral. Strata in each reservoir were dated through a combination of accelerator mass spectroscopy (AMS) radiocarbon (^14^C) dates (Supplementary Table S1) and ceramic seriation. Samples from 35 different strata from four reservoirs were analyzed.

## Results

**Figure 2:**
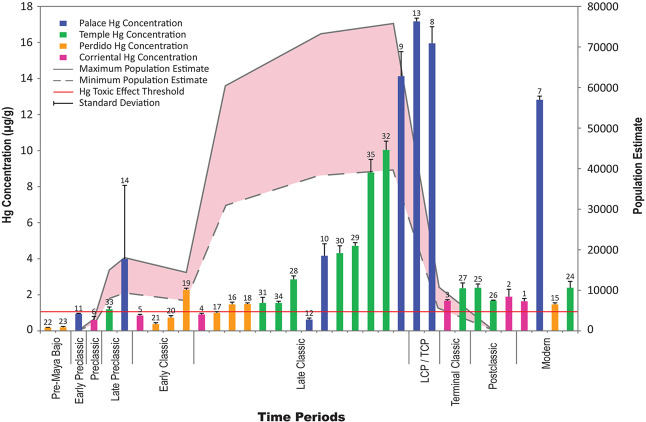
Mercury readings from four major reservoirs with the Tikal population curve^2^ in the background. Peak mercury levels coincide with the Late Classic/Terminal Classic period population apogee just prior to the abandonment of Tikal. Sample numbers are listed above each bar. Supporting data can be found in Supplementary Table S2.

Geochemical assays, using atomic absorption spectrophotometry following catalytic decomposition and gold amalgamation^17^, revealed high levels of mercury (Hg) in sediments associated with Late (600–830 CE) and Terminal Classic (830–900 CE) time periods (Supplementary Fig. S1) in both the Temple and Palace Reservoirs. Of the samples analyzed, 25 showed Hg levels that exceeded the toxic effect threshold (TET), the level above which the sediments are deemed to be heavily polluted^18^. Ten samples had levels below the TET (1.0 µg/g). One sample from the Temple Reservoir and four samples from the Palace Reservoir (Fig. 2) had Hg levels exceeding ten times the TET concentrations for sediments in freshwater ecosystems^18^. Four of the five samples with extremely high Hg readings were from strata associated with the Late or Terminal Classic periods, the time just before the abandonment of the site.

Hg was targeted for analysis because of its deleterious health effects. Also, the ancient Maya are known to have used mercury compounds, especially cinnabar (HgS) which has a blood-red color, in their dyes and paints for artifact decoration, architectural ornamentation^19^ and in ritual activities^20^. Virtually all of the elite burials at Tikal contained copious amounts of cinnabar^21^. For example, elite Burial 160 was enveloped by approximately 10 kg of powdered cinnabar^22^. In addition, liquid Hg has been discovered at numerous Maya sites, usually in vessels associated with ceremonial caches and burials^22,23^.

Mercury used in these ways by the ancient Maya likely was the source of the elevated readings observed at Tikal. Numerous buildings and plazas, mostly built during the Late Classic period, including the ceremonial burial zone of the North Acropolis, two ball courts, four major temples and two huge palace complexes (the Central and South Acropoli) all shed runoff into the Temple and Palace Reservoirs (Fig. 1). Because these reservoirs were located at the epicenter of the ceremonial and elite residential complex of Tikal, there were ample opportunities for Hg to wash into them. This circumstance created a huge potential health hazard for anyone who consumed their waters. Another possible source of mercury would have come from particulate matter from volcanic eruptions^24^. If this was the only source of mercury in the reservoirs at Tikal, however, we would expect a more random distribution of the metal at least spatially if not chronologically.

**Figure 3:**
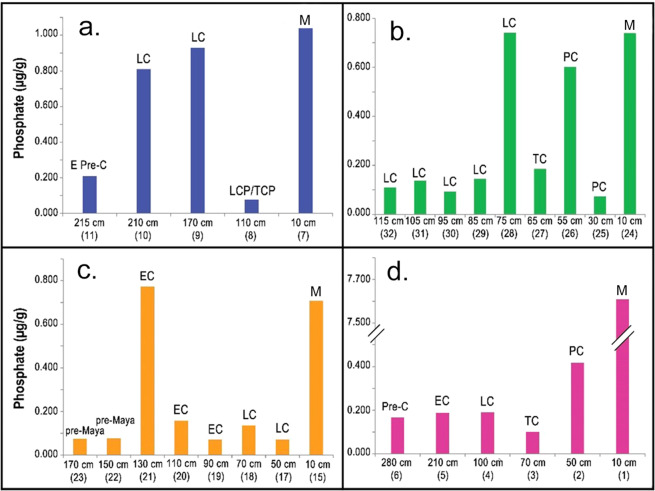
Phosphate readings, a proxy indicator for organic pollution, from four reservoirs at Tikal, viz., (**a**). Palace, (**b**). Temple, (**c**). Perdido, and (**d**). Corriental. Abbreviation key: Pre-Maya = Pre-Maya period; E Pre-C = Early Preclassic period; Pre-C = Preclassic period; EC = Early Classic period; LC = Late Classic period; TC = Terminal Classic period; PC = Postclassic period; M = modern. Supporting data can be found in Supplementary Table S3.

Phosphate (PO_4_^3−^) concentrations also increased in the Temple and Palace Reservoirs during the Late and Terminal Classic periods. Phosphate readings are often used as a proxy for increased organic deposition at archaeological sites because it is associated with human activities such as food waste discard and fecal contamination. Phosphate binds with soil and remains stable for many centuries^25^. In the Palace Reservoir, phosphate readings for the Late Classic period (0.80–0.92 µg/g) revealed a fourfold increase from the earlier Preclassic period (0.20 µg/g) (Fig. 3). Organic contamination likely came from the kitchen built on the water’s edge of the Palace Reservoir^21^ where meals were prepared for the elite residents of the Central Acropolis which is located just north of the Palace Reservoir. Hundreds of years of smoky cooking fires and ceramic plates washed in the reservoir added organic material to the waters. To make matters worse, the Maya cooks apparently dumped food wastes right outside of the kitchen, as evidenced by the presence of an adjacent midden^26^. During the rainy seasons, effluent from this trash pile would have washed directly into the reservoir.

Temple Reservoir also had a high phosphate peak in Late Classic sediments (0.72 µg/g) compared to earlier deposits (0.11–0.14 µg/g). Phosphate readings from Perdido were mostly quite low except for an Early Classic period spike (0.77 µg/g) in a sub-floor level that served as the foundation for the Early Classic plaster floor of the reservoir (Fig. 3). All of the Maya era phosphate readings were relatively low in the Corriental Reservoir. This is consistent with this reservoir being more distant from of the site core and evidence that it was likely kept clean by a set of sand and zeolite-containing boxes that served to filter the incoming water^13,14,27^. All of the reservoirs have high phosphate readings on the surface due to the formation of modern, organic-rich top-soils, enriched by dust and ash trapped by the forest regrowth^3,28^ since Tikal was abandoned.

**Figure 4:**
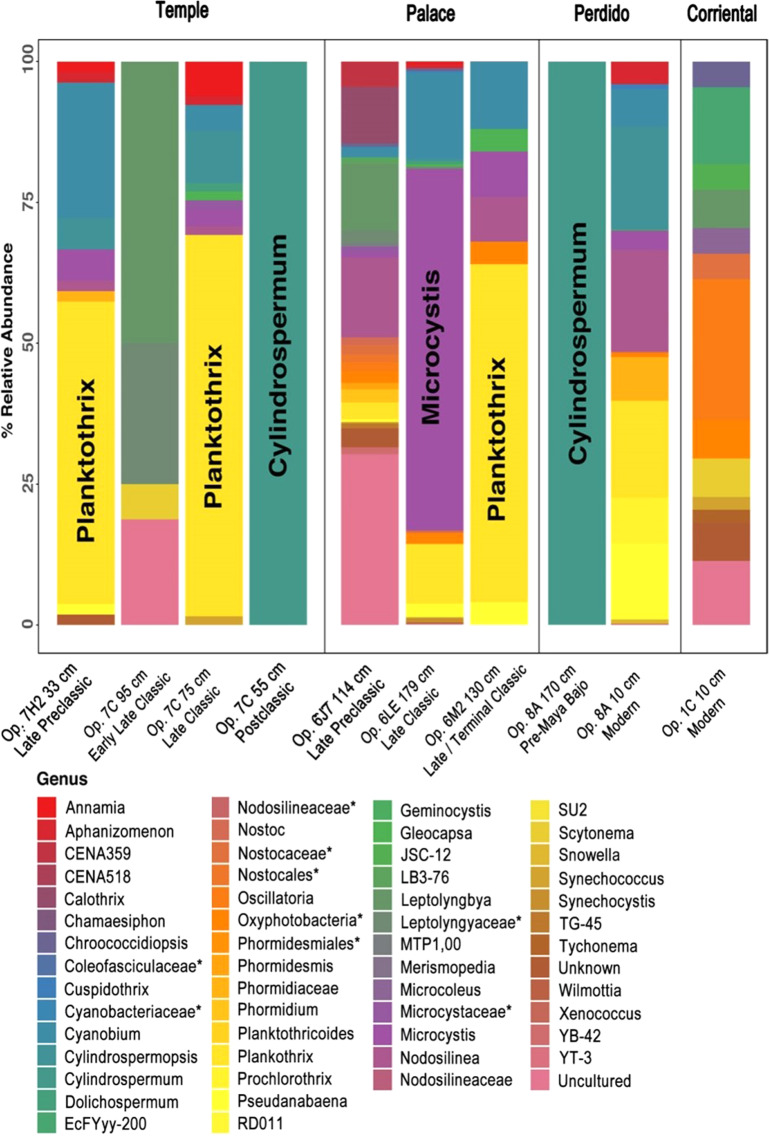
Cyanobacteria molecular genetic results from four reservoirs at Tikal. Evidence of cyanobacteria, or blue-green algae, blooms of *Planktothrix* and *Microcysitis* (both known to produce highly toxic microcystins) were found in the Temple and Palace Reservoirs. Taxa marked with an “*” denote unclassified strains. Supporting data can be found in Supplementary Table S4.

16S rRNA amplicon sequencing from sediment samples indicated large populations of problematic cyanobacteria (blue-green algae) in the Temple and Palace Reservoirs^29^ (Fig. 4). *Planktothrix* NIVA-CYA15 was the dominant cyanobacterium in Late Preclassic (300 BCE-200 CE) and Late Classic deposits recovered from the Temple Reservoir as well as Terminal Classic deposits from the Palace reservoir. In slightly earlier Late Classic strata from the Palace Reservoir, a *Microcystis* PCC-7914 proliferation was observed. *Planktothrix* and *Microcystis*, both planktonic cyanobacteria, are known to produce deadly toxins^29^. Therefore, our results indicate toxigenic blue-green algal blooms in two of the main reservoirs just prior to the time of abandonment in the mid-9^th^ century CE as a period of severe dryness set in. Similarly, a *Planktothrix* bloom occurred in the Temple Reservoir during the Late Preclassic, an earlier time period that also was plagued by droughts^30–32^. Microcystin toxins produced by *Planktothrix* and *Microcystis* can cause detrimental health effects even at extremely low concentrations (~ 2 nM), and are resistant to boiling^33^. With the presence of microcystins in Tikal’s reservoirs, the water could not have been safely consumed.

A third strain of cyanobacteria, *Cylindrospermum* NQAIF308^34^, was identified in high percentages in pre-Maya basal soil under the Perdido reservoir and in post-abandonment sediments in Temple Reservoir. *Cylindrospermum* NQAIF308 is common in terrestrial ecosystems, especially in soil^35^. *Cylindrospermum* likely was present before the reservoirs were built and after sediment filled the abandoned reservoirs as they dried up and the process of soil formation ensued.

## Discussion

Several key points emerge that provide a fine-grained picture of the end of the Maya occupation of Tikal. First, the main water sources for the site core of Tikal, especially the Temple and Palace Reservoirs, were seriously compromised as sources of drinking water by the end of the Late Classic period. This period of reduced rainfall co-occurred with expanded Tikal populations^2^ when they were most reliant on water reserves.

The drinking and cooking water for the Tikal rulers and their elite entourage almost certainly came from the Palace and Temple Reservoirs. As a result, the leading families of Tikal likely were fed foods laced with mercury at every meal. Contaminated waters would have had a negative impact on the health of the community, especially the ruling elite, and may have compromised their ability to lead effectively. Although the physiological mechanisms are not clear, there is a significant interrelation between chronic mercury exposure and aspects of metabolic syndrome, obesity in particular^36^. “Dark Sun”, a Terminal Classic Tikal ruler who was notably obese (Supplementary Fig. S2), may have suffered from this syndrome.

In the droughts of the 9^th^ century, the eutrophic, stagnant and receding waters of the Palace and Temple Reservoirs, with high levels of phosphate, would have been a perfect medium for the proliferation of cyanobacteria. The other reservoirs, especially Corriental and to a lesser extent Perdido, seem to have had fewer contamination problems. Corriental didn’t have drainage coming directly from the large temples, palaces, ballcourts or their surrounding plazas, and water flowing into the reservoir was probably filtered^13,14^. We have no evidence that Perdido Reservoir experienced cyanobacteria contamination although there was some influx of mercury. This reservoir was supplied by water through a series of canals from the “Lost World,” a ceremonial complex dating to the Preclassic and Early Classic periods^37^, but it was over 0.5 km from the source of possible mercury contaminants. Previous studies indicate that this reservoir was likely used for crop irrigation^4,14^ in addition to its possible use as a source of drinking water.

The authority of Classic Maya rulers was strongly linked to the provision of clean water^38^ among other things. There may well have been those who saw the events described above and the concomitant droughts as a failure of their leaders to adequately appease the Maya gods. Indeed, these events coming together must have resulted in a demoralized populace who, in the face of dwindling water and food supplies, became more willing to abandon their homes.

The final demise of Tikal was a complex tapestry of interwoven calamities. The results presented herein are complementary to hypotheses of climatic aridification and environmental degradation. Our findings provide synchronous, compounding factors occurring during the Late/Terminal Classic period, such as the contamination by mercury, organic pollutants and blue-green algal blooms. What has been observed at Tikal was a deadly combination of factors that worked together during the Terminal Classic period to make a continued existence at the city unsustainable. Furthermore, this scenario likely played out at other Maya cities dependent on reservoirs and this study presents new methodologies that can be used to test this hypothesis elsewhere.

The conditions described in this paper help to explain why Tikal, and possibly other sites in the Maya Lowlands were abandoned and why the sites were not re-occupied after the droughts of the 9^th^ century came to an end. The conversion of Tikal’s central reservoirs from life-sustaining to sickness-inducing places would have both practically and symbolically helped to bring about the abandonment of this magnificent city.

## Methods

Sediment samples were collected during excavations in 2009 and 2010 from strata within four Tikal reservoirs. To avoid modern contamination, as well as cross contamination from one sample to the next and from one stratum to the next, we took a column of samples^39^ from a characteristic side wall in each excavation unit or pit. This was completed by shaving the wall with a clean, sharp trowel where the sample was to be extracted. Sample collection was accomplished by starting at the bottom of the pit and working upwards in 10 cm increments. Sediment for the pollen, molecular and geochemical samples were removed with a clean trowel from the freshly shaved wall surface and placed in sterile plastic bags (Whirl-Packs) and labeled. Separate flotation samples (2 litres each) were collected at the same time giving us the benefit of micro-remain and macro-remain plant data from each context. The macro-remain samples were processed by water flotation^39^ and the pollen samples were sent to a laboratory in the US for pollen extraction. At the pollen lab, 2 g of sediment was removed from each sample with a sterile spatula then the bags were immediately resealed. Results of the pollen and macrobotanical analyses have been published previously^4,13,40,41^.

After arrival at the University of Cincinnati Paleoethnobotanical Laboratory, three 5-gram aliquots were removed from each sediment sample bag and inserted into sterile containers using sterile spatulas. Samples designated for molecular genetic analysis were placed in a −80 °C freezer until processing could begin. Just prior to DNA extraction, samples were thawed to −4 °C then inserted, under sterile conditions, into tubes with glass beads then sealed prior to homogenization in a bead-beating machine. Samples designated for phosphate tests were stored at room temperature then air dried and sieved using a clean 2 mm mesh sieve prior to analysis. Samples scheduled for mercury tests also were stored at room temperature then air dried and thermally and chemically decomposed within a decomposition furnace prior to analysis.

### Geochemical analyses

Total mercury in sediment samples was determined by atomic absorption spectrophotometry following catalytic decomposition and gold amalgamation using a Milestone Direct Mercury Analyzer (DMA-80, Shelton, CT, USA). This procedure follows standard protocols established by the US Environmental Protection Agency for the measurement of mercury in freshwater ecosystems^17^. The detection limit for the instrument is approximately 1 microgram per kilogram of sample material. The relative precision determined from triplicate analysis of a 10 ng g^−1^ and 100 µg g^−1^ reference was less than 10%. The method detection limit (MDL), defined as 3.143 times the standard deviation of seven blanks, was 0.013 ng g^−1^. The method reporting limit (MRL) defined as 3.143 times the standard deviation of seven reference samples was 0.099 ng g^−1^. Approximately 0.02 g of sediment was weighed out into a nickel plate quartz sample cell using a 0.1 mg balance and the mass was recorded. Samples were initially heated to 200 °C for 70 minutes and then heated at 650 °C for 3 minutes for thermal decomposition. Samples were amalgamated for 12 seconds and analyzed by atomic absorption for 30 seconds. Analyses of each sample were conducted in triplicate and results are presented in Fig. 2 and Supplementary Table S2.

Phosphate tests were conducted on Tikal reservoir sediment samples at the Service Testing and Research (STAR) Laboratory of the Ohio State University (Wooster, OH, USA). Sediment samples (2 g each) were analyzed using the Bray P1 procedure. Because the Tikal sediment samples all had pH values between 7 and 8, we determined that the Bray P1 procedure would be appropriate for phosphate analysis. The initial test extractant was a 0.025 normal hydrochloric acid (HCl) solution followed by an ammonium fluoride solution (0.03 normal NH_4_F). The extracted results were analyzed by a calibrated probe colorimeter set at 880 nm. Available phosphorus was determined by measuring the blue color intensity in solution when treated with reagent grade molybdate-ascorbic acid according to methods described by Kuo^42^. Results were reported in the form of available P or PO_4_^3−^ in μg/g and are presented in Fig. 3 and Supplementary Table S3.

### Molecular genetic studies

16S rRNA genes were sequenced using the paired-end Illumina MiSeq sequencing platform to characterize the community composition across temporal and spatial gradients throughout four Tikal reservoirs. Our preliminary characterization of the samples resulted in the extraction of DNA of sufficient quality for Illumina MiSeq sequencing and our data indicated that surface and subsurface assemblages were distinct (Supplementary Fig. S3).

DNA was extracted from sediment samples (n = 35) as described previously^43^. Each extract was sequenced using primers for amplification of the V4 region of the 16S rRNA (515F/805R) gene using the paired-end Illumina MiSeq sequencing platform. Sequence analysis and community ecology analysis were carried out using methods published previously^31^. This method has been employed to successfully extract high quality DNA from a subset (n = 12) of samples from Tikal and characterize the community composition of those samples. In addition, since ancient DNA tends to be fragmented and could pose a challenge for this study, several techniques to isolate ancient DNA were evaluated^44–46^.

The DNA samples also were subjected to taxa-specific quantitative PCR (qPCR). A recently developed suite of methods aimed at determining fecal pollution in environment samples has been developed and we followed those protocols^47^. To guide these studies, we searched for specific taxa in the community composition data acquired and analyzed as described above. In samples that contained taxa of interest, we employed taxa-specific primers to determine changes in abundance of specific taxa across the temporal and spatial gradients throughout the Tikal landscape. For instance, our targets included (but were not limited to) toxin producing cyanobacteria. qPCR was performed and assayed on a StepOne Plus real-time PCR instrument from Invitrogen.

All reactions were completed in triplicate using the optimal conditions determined above. Specificity of the qPCR assays were verified by melt curve analysis. For qPCR, we performed qPCR analysis on a subset of samples (n = 50). Each run of the assays included positive and negative controls resulting in ~500 total assays. Following quality control, merging of contigs, and removal of chimeras and singletons, we recovered 663,817 16S rRNA sequences with an average length of 253 bp. The libraries ranged in size from 9,421 to 49,863 total sequences following quality control. At a sequence identity of 97%, we recovered 354 Operational Taxonomic Units (OTUs) affiliated with archaea and 20,212 OTUs affiliated with bacteria.

In the Temple Reservoir we recovered cyanobacterial OTUs (Fig. 4) from a Late Preclassic period sample (sample #45), an Early Late Classic (#30), a Late Classic (#28), and a Postclassic sample (#26). Sequences recovered from the Postclassic horizon were affiliated with the Nostocaceae (*Cylindrospermum* sp.). OTUs recovered from the Early Classic and Late Classic were affiliated with uncultured Leptolyngbyaceae, and Nostocaceae. Phormidiaceae (*Plankothrix* sp.) were abundant in the Late Classic and Late Preclassic samples where these OTUs accounted for >60% of the total Oxyphotobacteria sequences. We also recovered OTUs affiliated with Cyanobiaceae, Nostocaceae, and Leptolyngbyaceae from the Late Classic and Late Preclassic samples.

In the Palace Reservoir, we recovered cyanobacterial OTUs from Late Preclassic (#14), Late Classic (#12), and the Late/Terminal Classic period (#13). OTUs affiliated with Cyanobiaceae were recovered from all three samples. OTUs affiliated with Microcystaceae (*Microcystis* sp.) were abundant in the Late Classic samples. OTUs affiliated with Phormidiaceae (*Plankothrix* sp.) were the most abundant cyanobacterial sequences recovered from the Late Classic/Terminal Classic samples.

From the Perdido Reservoir we recovered cyanobacterial OTUs from pre-Maya bajo samples (#23, 170 cm below surface) and the A1 horizon (#15, 10 cm below surface). All OTUs recovered from the pre-Maya bajo sample were affiliated with Nostocaceae. OTUs affiliated with Nostocaceae (*Cylindrospermosum* sp.) were also recovered from the A1 sample along with Nodosilineaceae, Cyanobiaceae, Microcystaceae (*Microcystis* sp.), Phormidiaceae, Prochlorotrichaceae, and Pseudanabaenaceae. In the Corriental Reservoir, only a surface sample (#1) with cyanobacterial OTUs contained gene sequences affiliated with uncultured Leptolyngbyaceae (*Oscillatoria* sp.). Results of the molecular genetic assays involving cyanobacteria are illustrated in Fig. 4 and additional details are provided in the Supplementary Information section and the Supplementary Table S4.

## Supplementary information

Supplementary information.

## Data Availability

Raw sequence reads for the genetic analyses are available on the National Center for Biotechnology Information (NCBI) Short Read Archive (SRA) under the BioProject number PRJNA579387. All other data are available in the manuscript or in the Supplementary Information section.
