# Supplementary material for: Molecular genetic and geochemical assays reveal severe contamination of drinking water reservoirs at the ancient Maya city of Tikal
Source: Sci Rep. 2020 Jun 25;10:10316. doi: 10.1038/s41598-020-67044-z (PMC7316703; doi:10.1038/s41598-020-67044-z)
Supplement: Supplementary file 1 — Supplementary information. [file 41598_2020_67044_MOESM1_ESM.pdf]

Molecular genetic and geochemical assays reveal severe contamination of drinking water reservoirs at the ancient Maya city of Tikal

Authors
David L. Lentz, Trinity L. Hamilton, Nicholas P. Dunning, Vernon L. Scarborough, Todd P. Luxton, Anne Vonderheide, Eric J. Tepe, Cory J. Perfetta, James Brunemann, Liwy Grazioso, Fred Valdez, Kenneth B. Tankersley, Alison A. Weiss

Community composition and genetic diversity

In terms of alpha diversity, DNA samples from Palace and Temple Reservoirs at Tikal had the highest diversity at the OTU and genus levels compared to the Corriental and Perdido Reservoirs. Alpha diversity in the Perdido Reservoir samples showed a large range including the lowest diversity observed in all samples. At the Phylum level, the majority of OTUs recovered from all the sites were affiliated with Actinobacteria, Proteobacteria, Chloroflexi and Acidobacteria. Smaller numbers of OTUs affiliated with Planctomycetes, Robubacteria, Thaumarcheaota and Verrucomicrobia were also observed (Supplementary Fig. S4).

Following our molecular analyses, at the Phylum level, the majority of OTUs recovered from all the sites were affiliated Actinobacteria, Proteobacteria, Chloroflexi and Acidobacteria. Smaller numbers of OTUs affiliated with Planctomycetes, Robubacteria, Thaumarcheaota and Verrucomicrobia were also observed. At the Class level (Supplementary Fig. S5), the majority of OTUs recovered from the surface samples and Temple and Palace Reservoirs were affiliated with Actinobacteria, Alphaproteobacteria, Subgroup 6 and Thermoleophilia. OTUs affiliated with Thermoleophilia accounted for > ~20-25% of all sequences in these samples. In contrast, OTUs affiliated with Actinobacteria and Alphaproteobacteria accounted from >50% of the total sequences in most of Preclassic and Early Classic Period Perdido Reservoir samples. The two exceptions were the Late Classic and the Late Preclassic samples— Thermoleophilia was also abundant in these samples. In the Perdido Reservoir samples, Alphaproteobacteria and Actinobacteria were the most abundant. Smaller numbers of Acidomicrobiia, Subgroup 6, MB_A2_108, and NC10 were observed in all the samples. Sequences affiliated with Bacilli were also abundant in the Palace Reservoir samples. We recovered OTUs affiliated with Oxyphotobacteria (phototrophic Cyanobacteria) from a subset of the samples (Fig. 4).

Supplementary Fig. S1. Chronology of Tikal. The Gregorian column represents the recording of time according to the modern Christian calendar and the Long Count represents the Maya calendar. The site core of Tikal was abandoned during the Terminal Classic period. Time periods follow Houston and Inomata[1].

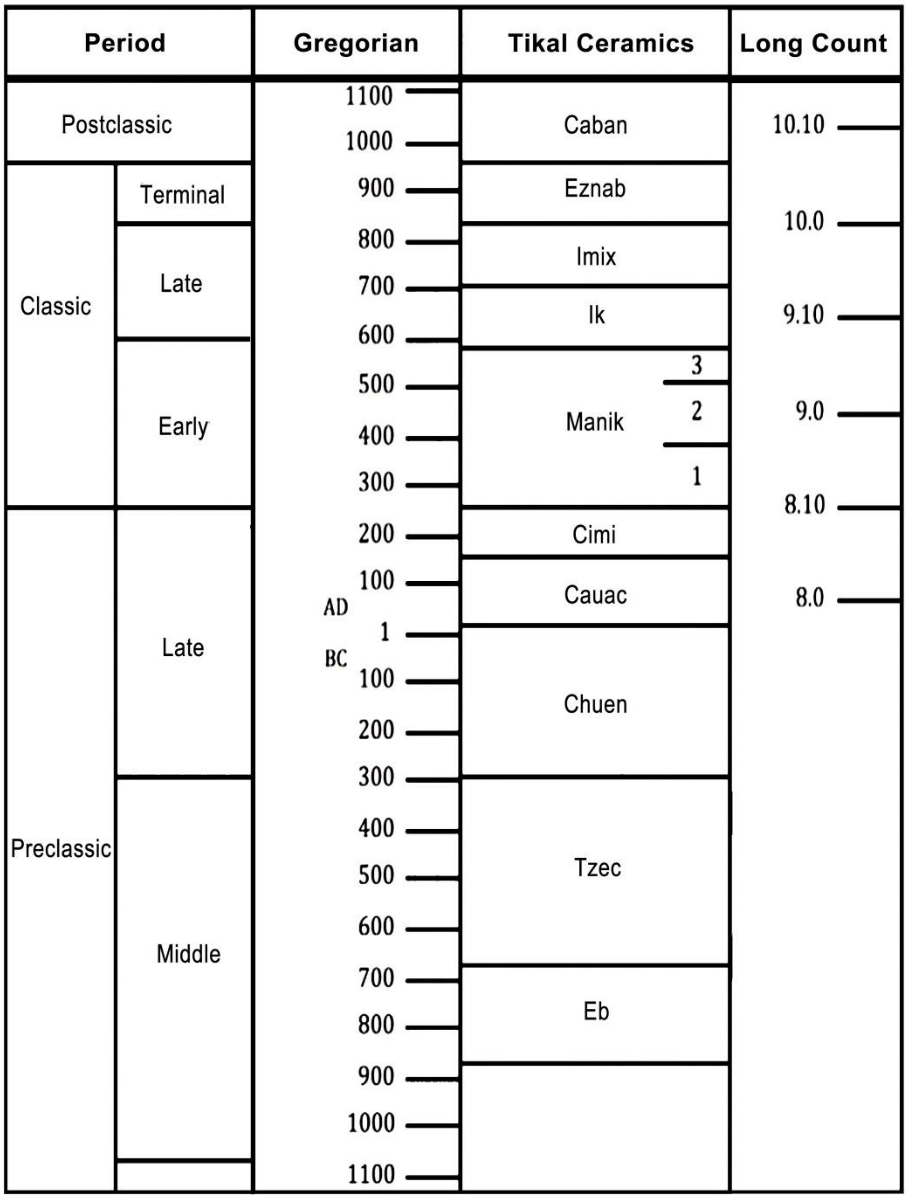

Supplementary Fig. S2. One of the last rulers of Tikal, Dark Sun, may have suffered from metabolic syndrome related to chronic mercury poisoning. Obesity is one of the side effects of the syndrome. This image was drawn by William R. Coe from the carved surface of Lintel 2 in Temple III (Courtesy of the Penn Museum, image no. Tikal 57-5-7).

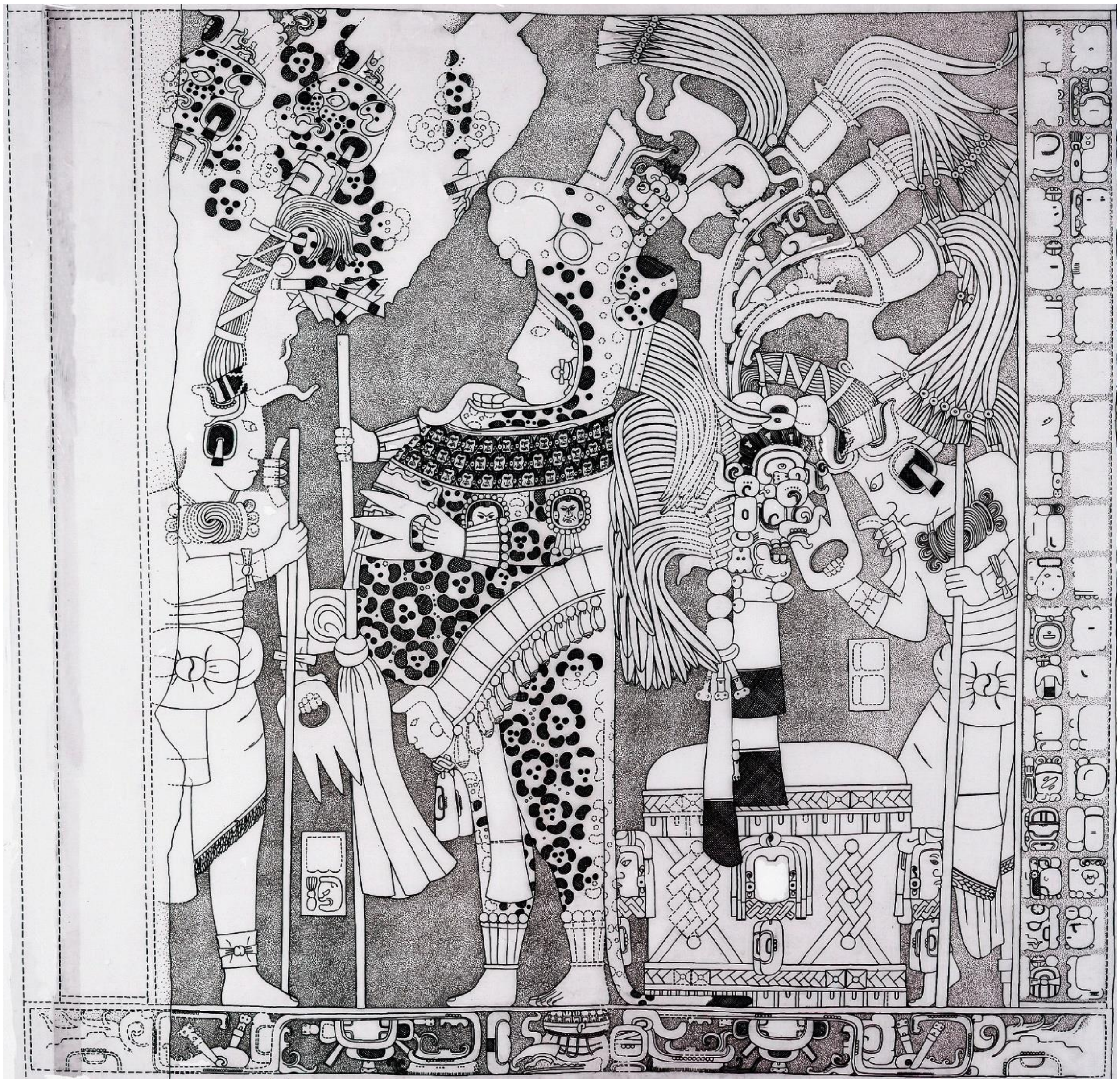

Supplementary Fig. S3. A PCoA analysis of Bray-Curtis similarities indicate that microbial communities from the surface were distinct from those of the Precolumbian strata of the Temple and Palace Reservoirs.

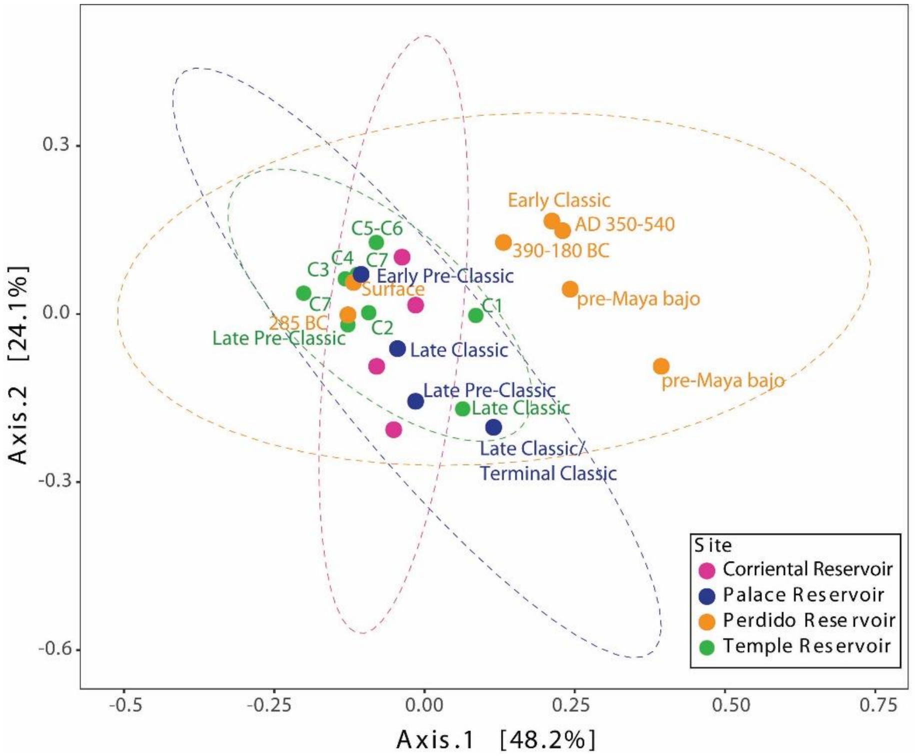

Supplementary Fig. S4. Community composition and diversity. Alpha diversity metrics of 16S rRNA gene sequences recovered from Tikal surface and reservoir sediments. Samples were grouped by reservoir.

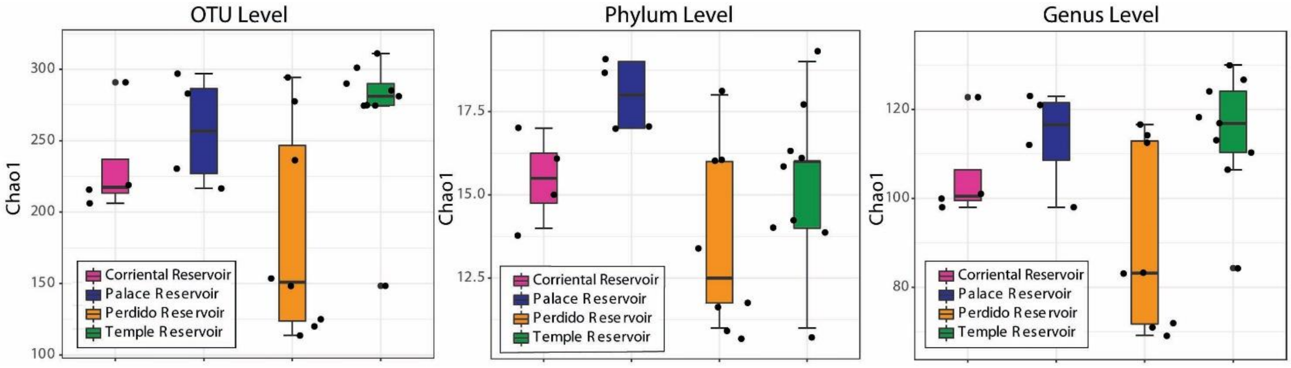

Supplementary Fig. S5. Composition of 16S rRNA gene sequences affiliated with bacteria recovered from the reservoir and surface samples. Representative OTUs for each library were binned at the Class level. From this set of taxa, only phototrophic cyanobacteria were included in Fig.4.

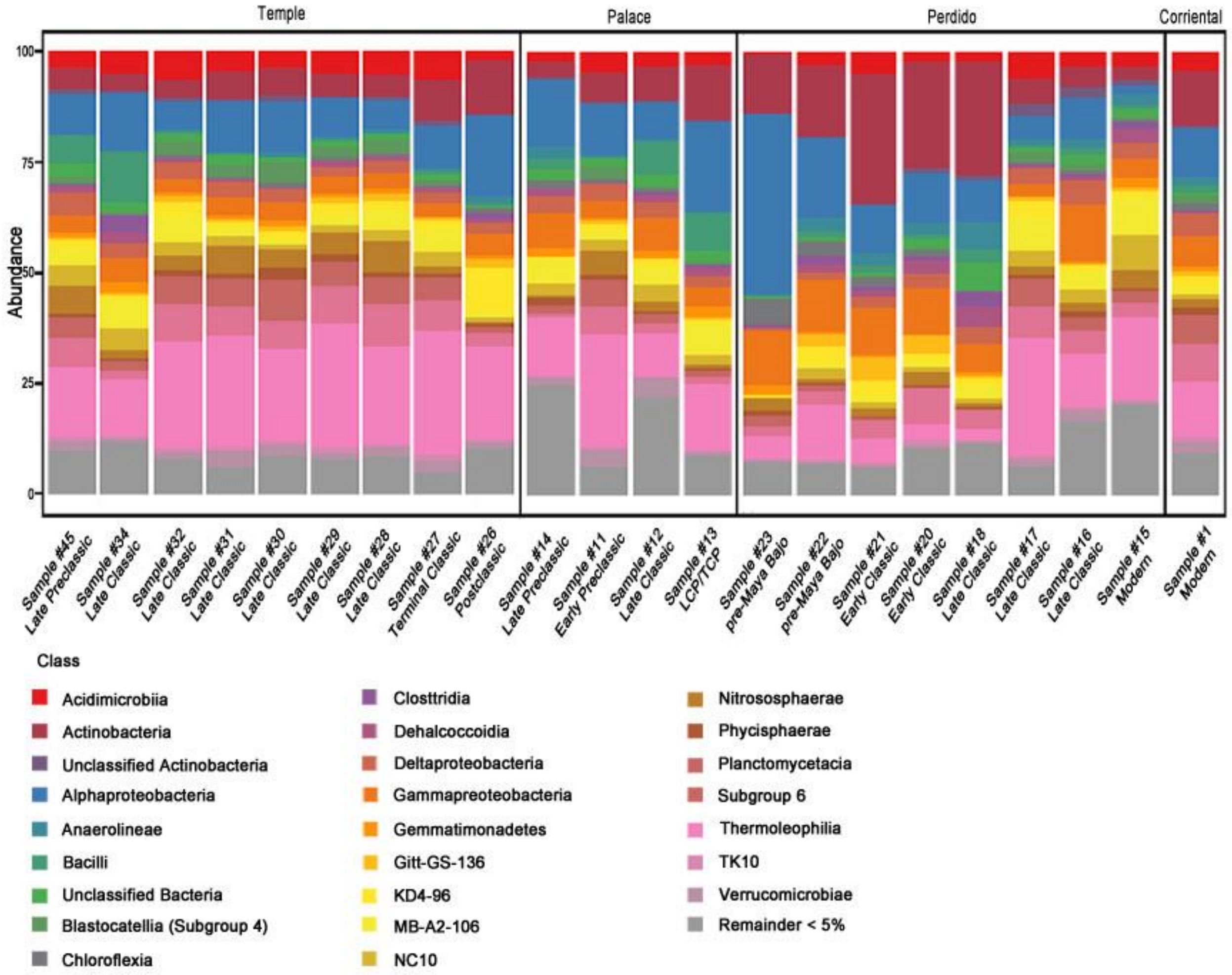

Supplementary Table S1. Accelerator mass spectrometry (AMS) radiocarbon (^{14}C) dates for Tikal reservoirs and related contexts discussed in this text. AMS dates were obtained from Beta Analytic (Miami, FL, USA) and the National Ocean Sciences Accelerator Mass Spectrometry Facility (Woods Hole, MA, USA). Data provided include AMS radiocarbon sample composition (SOM = soil organic matter), provenience, depth in cm, measured radiocarbon years before present (BP), and calibrated age at two sigma margins for error. Samples were collected from wet and dry cores and excavation profiles. These data were extracted from larger tables published previously[2,3].

Lab Number	Composition	Reservoir	Provenience	Depth (cm)	Measured ^{14}C (yr BP)	Calibrated Age (2 σ)
Beta-258720[a]	Charcoal	Corriental	Op 1C	65-80	990 ± 40	1010-1170 CE
Beta-280839[b]	SOM	Corriental	Op 1L: Core 8	140-180	2,010 ± 40	340-30 BCE
Beta-266124[a]	SOM	Corriental	Op 1C	162-194	2,110 ± 40	190-80 BCE
Beta-280837[b]	SOM	Corriental	Op 1L: Core 8	180-230	2,120 ± 40	380-170 BCE
Beta-258721[a]	SOM	Corriental	Op 1C	265-290	2,340 ± 40	760-400 BCE
Beta-270566[b]	SOM	Corriental	Op 1L: Core 8	310-312	8,960 ± 60	8290-7970 BCE
Beta-266122[a]	SOM	Corriental	Arroyo Op 2A	90	2,110 ± 40	190-80 BCE
Beta-274990[b]	Charcoal	Corriental	Berm, Op 1L: Core 17	Anthrosol	1,560 ± 40	400-570 CE
Beta-266123[a]	SOM	Corriental	Pocket Bajo 1, OP 2b	60	1,930 ± 40	90 BCE-80 CE
88675[b]	SOM	Corriental	Pocket Bajo 2, Op 1L: Core 21-1	50-60	6,250 ± 35	5312-5076 BCE
Beta-281750[a]	Charcoal	Palace	Op 6Q	Above Dam Collapse	1,250 ± 40	670-880 CE
Beta-281751[a]	Charcoal	Palace	Op 6Q	Below Dam collapse	1,260 ± 40	660-880 CE
Beta-288914[a]	Charcoal	Palace	Op 6L	150	1,380 ± 40	610-680 CE
Beta-281745[a]	SOM	Palace	Op 6O	Channel fill	3,310 ± 40	1870-1850 BCE

Table S1. Continued**.**

88638[a]	SOM	Palace	Op 6J-13: Core 1-1	50-60	3,360 ± 30	1739-1535 BCE
88682[a]	SOM	Palace	Op 6J-13: Core 2	100-110	2,150 ± 40	358-55 BCE
Beta-281747[a]	Charcoal	Perdido	Op 8K	49-54	6,810 ± 40	5740-5640 BCE
Beta-289287[b]	SOM	Perdido	OP 8: Core N2E0	50-60	2,220 ± 60	390-180 BCE
Beta-280828[a]	SOM	Perdido	Op 8A	110	1,540 + 40	350-540 CE
Beta-289286[b]	SOM	Perdido	OP 8: Core N2E0	180-190	15,110 ± 60	16860-16740 BCE
Beta-289285[b]	SOM	Perdido	OP 8: Core N2E0	280-290	15,480 ± 60	16920-16780 BCE
Beta-289284[b]	SOM	Perdido	OP 8: Core N2E0	390-395	15,310 ± 60	16820-16670 BCE
Beta-281748[a]	Charcoal	Perdido	Pocket Bajo, Op 8D	76-79	1,570 ± 40	400-570 CE
Beta-281746[a]	Charcoal	Temple	Main Tank, Op 7C	110	1,200 ± 40	680-890 CE
85584[a]	SOM	Temple	Main Tank, OP 7C	130-140	1,230 ± 25	721-839 CE
85585[a]	SOM	Temple	Main Tank, OP 7C	140-162	1,830 ± 25	143-215 CE
85583[a]	SOM	Temple	Main Tank, OP 7C	162-194	1,250 ± 35	701-811 CE
88676[b]	SOM	Temple	Silting Tank, Op 7: Core 23-2	70-80	195 ± 35	1645-1952 CE
88677[b]	SOM	Temple	Silting Tank, Op7: Core 23-2	110-120	2,330 ± 40	521-216 BCE
Beta-298985[a]	Charcoal	Temple	Silting Tank, OP7A	130	1,370 ± 30	640-680 CE

a. Sample collected from excavation profile;
b. Dry core with some compression.
c. Wet core with compression.

Supplementary Table S2. Mercury concentration table. Results shown are displayed in Figure 2.

ID	Reservoir and Operation	Depth (cm)	Context	Hg Concentration (μg/g)				SD	RSD
				Rep. 1	Rep. 2	Rep. 3	Avg. of Sample Rep.		
1	Corriental: 1C	10	Modern	1.72	1.47	1.74	1.643	0.15	9%
2		50	Postclassic	1.608	2.42	1.6	1.89	0.46	24%
3		70	Terminal Classic	1.73	1.71	1.70	1.71	0.02	1%
4		100	Late Classic	1.00	0.87	0.84	0.90	0.08	9%
5		210	Early Classic	0.81	0.94	0.80	0.85	0.08	9%
6		280	Preclassic	0.83	0.42	0.41	0.55	0.24	43%
7	Palace: 6L	10	Modern	12.32	12.87	13.21	12.80	0.45	4%
8		110	LCP/TCP	14.96	16.95	15.94	15.95	1.00	6%
9		170	Late Classic	13.35	15.83	13.21	14.13	1.47	10%
10		210	Late Classic	5.00	3.68	3.75	4.15	0.74	18%
11		215	Early Preclassic	0.94	0.96	0.93	0.94	0.02	2%
12	6L E-Profile	179	Late Classic	0.73	0.63	0.54	0.63	0.10	15%
13	6M	130	LCP/TCP	17.27	17.28	16.92	17.16	0.21	1%
14	6J	114	Late Preclassic	2.23	8.68	1.05	3.99	4.11	103%
15	Perdido: 8A	10	Modern	1.40	1.57	1.41	1.46	0.10	7%
16		30	Late Classic	1.34	1.65	1.41	1.46	0.16	11%
17		50	Late Classic	1.05	0.99	0.97	1.01	0.04	4%
18		70	Early Classic	1.49	1.44	1.56	1.49	0.06	4%
19		90	Early Classic	2.22	2.25	2.39	2.29	0.09	4%
20		110	Early Classic	0.85	0.61	0.71	0.72	0.12	17%
21		130	Early Classic	0.31	0.28	0.49	0.36	0.11	31%
22		150	pre-Maya Bajo	0.16	0.16	0.21	0.18	0.03	16%

Table S2. Continued.

ID	Reservoir and Operation	Depth (cm)	Context	Hg Concentration (μg/g)					
				Rep. 1	Rep. 2	Rep. 3	Avg. of Sample Rep.	SD	RSD
23		170	pre-Maya Bajo	0.17	0.22	0.26	0.22	0.05	21%
24	Temple: 7C	10	Modern	1.97	2.61	2.64	2.41	0.38	16%
25		30	Postclassic	2.14	2.57	2.46	2.39	0.22	9%
26		55	Postclassic	1.73	1.56	1.64	1.65	0.08	5%
27		65	Terminal Classic	2.42	1.99	2.65	2.35	0.33	14%
28		75	Late Classic	3.09	2.74	2.72	2.85	0.21	7%
29		85	Late Classic	4.57	4.95	4.62	4.71	0.21	4%
30		95	Late Classic	3.96	4.12	4.83	4.30	0.46	11%
31		105	Late Classic	1.88	1.55	1.21	1.55	0.33	22%
32		115	Late Classic	10.14	10.52	9.49	10.05	0.52	5%
33		179	Late Preclassic	1.11	1.15	1.35	1.20	0.13	11%
34		95	Late Classic	1.63	1.57	1.46	1.55	0.08	5%
35		100	Late Classic	7.89	9.39	9.07	8.78	0.79	9%

Supplementary Table S3. Phosphate readings from four Tikal reservoirs. Results shown here are displayed in Figure 3.

ID	Reservoir	Provenance	Depth (cm)	Context	PO_4 (µg/g)
1	Corriental	1C 1	10	Modern	7.62
2		1C 5	50	Postclassic	0.417
3		1C 7	70	Terminal Classic	0.101
4		1C 10	100	Late Classic	0.190
5		1C 21	210	Early Classic	0.188
6		1C 28	280	Preclassic	0.166
7	Palace	6L 1b	10	Modern	1.04
8		6L 3b	110	LCP/TCP	0.077
9		6L 6b	170	Late Classic	0.930
10		6L 9b	210	Late Classic	0.809
11		6L 10b	215	Early Preclassic	0.210
15	Perdido	8A 1	10	Modern	0.708
17		8A 3	50	Late Classic	0.07
18		8A 4	70	Late Classic	0.136
19		8A 5	90	Early Classic	0.07
20		8A 6	110	Early Classic	0.157
21		8A 7	130	Early Classic	0.773
22		8A 8	150	pre-Maya bajo soil	0.076
23		8A 9	170	pre-Maya bajo soil	0.075
24	Temple	7C	10	Modern	0.740
25		7C	30	Postclassic	0.074
26		7C	55	Postclassic	0.602
27		7C	65	Terminal Classic	0.186
28		7C	75	Late Classic	0.741

Table S3. Continued

29	7C	85	Late Classic	0.145
30	7C	95	Late Classic	0.09
31	7C	105	Late Classic	0.137
32	7C	115	Late Classic	0.110

Supplementary Table S4. Cyanobacteria from four Tikal reservoirs. Abbreviations key: Late Pre-Cl = Late Preclassic period; Early Late Cl = Early Late Classic period; and Late/Term Cl = Late/Terminal Classic period. Names marked with an "*" denote an unclassified strain. Results shown are displayed in Figure 4.

ID#	Reservoir and Operation	Depth (cm)	Context	Family	Genus	Abun-dance	OTU
26	Temple: 7C	55	Postclassic	Nostocaceae	Cylindrospermum_NQAIF308	9	1922
28		75	Late Classic	Phormidiaceae	Planktothrix_NIVA-CYA_15	44	337
				Nostocaceae	Cylindrospermopsis_CRJ1	6	464
				Cyanobacteriaceae	Annamia_HOs24	4	4842
				Cyanobiaceae	Cyanobium_PCC-6307	3	1170
				Microcystaceae	Microcystis_PCC-7914	2	314
				Nodosilineaceae	Nodosilinea_PCC-7104	1	779
				Nostocaceae	Aphanizomenon_NIES81	1	1661
				Microcystaceae	Microcystaceae*	1	5691
				Gloeocapsaceae	Gleocapsa	1	6351
				Nostocaceae	Dolichospermum_NIES41	1	7506
				Cyanobiaceae	Synechococcus_MBIC10613	1	12481
30		95	Early Late Cl	unknown	Leptolyngbya_ANT.L52.2	8	1173
				Leptolyngbyaceae	Leptolyngbyaceae*	4	3312
				unknown family	uncultured	2	379
				Nostocaceae	Scytonema_PCC-7110	1	10262
				uncultured	uncultured	1	16266
45	Temple: 7H2	33	Late Pre-Cl	Phormidiaceae	Planktothrix_NIVA-CYA_15	29	337
				Cyanobiaceae	Cyanobium_PCC-6307	13	1170
				Microcystaceae	Microcystis_PCC-7914	3	314
				Nostocaceae	Cylindrospermopsis_CRJ1	3	464
				Pseudanabaenaceae	Pseudanabaena_PCC-7429	1	745
				Nodosilineaceae	Nodosilinea_PCC-7104	1	779
				Phormidiaceae	Phormidiaceae_ge	1	964
				Nostocaceae	Aphanizomenon_NIES81	1	1661
				unknown	unknown	1	2369
				Cyanobacteriaceae	Annamia_HOs24	1	4842
13	Palace: 6M2	130	Late/Term Cl	Phormidiaceae	Planktothrix_NIVA-CYA_15	15	337
				Cyanobiaceae	Cyanobium_PCC-6307	3	1170
				Microcystaceae	Microcystis_PCC-7914	2	314
				Nodosilineaceae	Nodosilinea_PCC-7104	2	779
				Pseudanabaenaceae	Pseudanabaena_PCC-7429	1	745
				Oxyphotobacteria*	Oxyphotobacteria*	1	5390
				Gloeocapsaceae	Gleocapsa	1	6351
12	Palace: 6LE	179	Late Classic	Microcystaceae	Microcystis_PCC-7914	325	314
				Cyanobiaceae	Cyanobium_PCC-6307	77	1170
				Phormidiaceae	Planktothrix_NIVA-CYA_15	54	337
				Pseudanabaenaceae	Pseudanabaena_PCC-7429	12	745
				Oxyphotobacteria*	Oxyphotobacteria*	10	5390
				Microcystaceae	Synechocystis_PCC-6803	4	10105
				Nostocaceae	Aphanizomenon_NIES81	3	1661
				Cyanobacteriaceae	Annamia_HOs24	3	4842
				Nostocaceae	Dolichospermum_NIES41	3	7506
				Nostocaceae	Cylindrospermopsis_CRJ1	2	464
				Leptolyngbyaceae	Leptolyngbyaceae*	2	3312
				Gloeocapsaceae	Gleocapsa	2	6351
				Cyanobacteriaceae	Cyanobacteriaceae*	2	10271
				Leptolyngbyaceae	Chamaesiphon_PCC-7430	2	12438
				Nodosilineaceae	Nodosilinea_PCC-7104	1	779
				unknown	unknown	1	2369
				Nostocaceae	Nostocaceae*	1	2983
				Microcystaceae	Snowella_0TU37S04	1	4612
				Leptolyngbyaceae	uncultured	1	5670
				Microcystaceae	Microcystaceae*	1	5691
				Nostocaceae	Nostoc_PCC-7524	1	12086
				Coleofasciculaceae	Coleofasciculaceae*	1	12117

Table S4. Continued.

ID#	Reservoir and Operation	Depth (cm)	Context	Family	Genus	Abun-dance	OTU
14	Palace: 6J7	114	Late Preclassic	unknown	uncultured	396	379
				Nodosilineaceae	Nodosilinea_PCC-7104	192	779
				unknown	Leptolyngbya_ANT.L52.2	154	1173
				Nostocaceae	Calothrix_PCC-6303	123	1411
				unknown	CENA359	58	2158
				unknown	unknown	45	2369
				Phormidiaceae	Planktothrix_NIVA-CYA_15	40	337
				Leptolyngbyaceae	Leptolyngbyaceae*	30	3312
				Phormidesmiaceae	Phormidium_MBIC10003	27	2383
				Oxyphotobacteria*	Oxyphotobacteria*	27	5390
				Nostocaceae	Nostocaceae*	24	2983
				Cyanobiaceae	Cyanobium_PCC-6307	23	1170
				Unknown	Oscillatoria_SAG_1459-8	22	2118
				Nostocales*	Nostocales*	19	4067
				Nostocaceae	Nostoc_PCC-73102	16	3489
				Leptolyngbyaceae	YB-42	15	3608
				Leptolyngbyaceae	Phormidesmis_ANT.L52.6	14	3665
				Microcystaceae	Microcystaceae*	14	5691
				Leptolyngbyaceae	LB3-76	13	3828
				Microcystaceae	Microcystis_PCC-7914	12	314
				Leptolyngbyaceae	TG-45	12	4126
				unknown	MTP1.00	8	5194
				Leptolyngbyaceae	uncultured	8	5670
				Nostocaceae	Calothrix_UAM_374	8	6905
				Chroococcidiopsaceae	Chroococcidiopsis_PCC_7203	7	6890
				unknown	Leptolyngbya_VRUC_135	5	6649
				Pseudanabaenaceae	Pseudanabaena_PCC-7429	4	745
				unknown	Calothrix_KVSF5	4	7393
				Pseudanabaenaceae	Synechococcus_PCC-7502	3	7846
				Leptolyngbyaceae	YT-3	3	8180
				unknown	Phormidium_CYN64	3	8405
				Chroococcidiopsaceae	uncultured	3	8734
				Nodosilineaceae	uncultured	3	12012
				Nostocaceae	Cylindrospermopsis_CRJ1	2	464
				Phormidiaceae	Phormidiaceae_ge	2	964
				Unknown	CENA518	2	9251
				Cyanobacteriaceae	Geminocystis_PCC-6308	2	10266
				Nodosilineaceae	Nodosilineaceae*	2	12017
				Unknown	Leptolyngbya_ANT.L67.1	2	16427
				Prochlorotrichaceae	Prochlorothrix_PCC-9006	1	1010
				Nostocaceae	Aphanizomenon_NIES81	1	1661
				Nostocaceae	Cuspidothrix_LMECYA_163	1	4374
				Cyanobacteriaceae	Annamia_HOs24	1	4842
				Microcystaceae	SU2_symbiont_group	1	11927
				Oscillatoriaceae	Planktothricoides_SR001	1	12026
				Coleofasciculaceae	Wilmottia_Ant-Ph58	1	12118
				Phormidesmiales*	Phormidesmiales*	1	12292
				Leptolyngbyaceae	Chamaesiphon_PCC-7430	1	12438
				Leptolyngbyaceae	Leptolyngbya_PCC-6306	1	12440
				RD011_fa	RD011_ge	1	16247
1	Corriental: 1C	10	Modern	unknown	Oscillatoria_SAG_1459-8	11	2118
				unknown	EcFYyy-200	6	6027

Table S4. Continued.

ID#	Reservoir and Operation	Depth (cm)	Context	Family	Genus	Abun-dance	OTU
1	Corriental 1C	10	Modern	unknown	uncultured	3	379
				unknown	Leptolyngbya_ANT.L52.2	3	1173
				unknown	Unknown*	3	2369
				Oxyphotobacteria*	Oxyphotobacteria*	3	5390
				Nostocaceae	Scytonema_PCC-7110	3	10262
				Nostocaceae	Nostocaceae*	2	2983
				Chroococcidiopsaceae	Chroococcidiopsis_PCC_7203	2	6890
				uncultured	uncultured_ge	2	10207
				Coleofasciculaceae	Microcoleus_SAG_1449-1a	2	10263
				Leptolyngbyaceae	JSC-12	2	11267
				Phormidiaceae	Tychonema_CCAP_1459-11B	1	11940
				Thermosynechococcaceae	Synechococcus_IR11	1	16248
15	Perdido: 8A	10	Modern	Nostocaceae	Cylindrospermopsis_CRJ1	221	464
				Nodosilineaceae	Nodosilinea_PCC-7104	218	779
				Phormidiaceae	Planktothrix_NIVA-CYA_15	209	337
				Pseudanabaenaceae	Pseudanabaena_PCC-7429	163	745
				Prochlorotrichaceae	Prochlorothrix_PCC-9006	100	1010
				Phormidiaceae	Phormidiaceae_ge	94	964
				Cyanobiaceae	Cyanobium_PCC-6307	82	1170
				Nostocaceae	Aphanizomenon_NIES81	46	1661
				Microcystaceae	Microcystis_PCC-7914	38	314
				Nostocaceae	Cuspidothrix_LMECYA_163	10	4374
				Microcystaceae	Snowella_0TU37S04	9	4612
				Oxyphotobacteria*	Oxyphotobacteria*	9	5390
				Microcystaceae	Microcystaceae*	4	5691
				Nostocales*	Nostocales_unclassified	2	4067
				unknown	uncultured	1	379
				Cyanobacteriaceae	Annamia_HOs24	1	4842
				Gloeocapsaceae	Gleocapsa	1	6351
				unknown	Calothrix_KVSF5	1	7393
				Nostocaceae	Dolichospermum_NIES41	1	7506
				Nodosilineaceae	Nodosilineaceae*	1	12017
				Microcystaceae	Merismopedia_0BB39S01	1	12084
				Nostocaceae	Aphanizomenon_MDT14a	1	12132
				Xenococcaceae	Xenococcus_CRM	1	12148
				Nodosilineaceae	Nodosilineaceae_ge	1	12436
				Pseudanabaenaceae	uncultured	1	13135
23		170	pre-Maya Bajo	Nostocaceae	Cylindrospermum_NQAIF308	28	1922

Supplementary References

1. Houston, S. D., & Inomata, T. *The Classic Maya* (Cambridge University Press, 2009).

2. Scarborough, V. L. *et al*., Water and sustainable land use in an ancient tropical city: Tikal, Guatemala. *Proc. Natl. Acad. Sci.* **109**, 12408-12413 (2012).

3. Lentz, D. L., Dunning, N. P., & Scarborough, V.L. (eds.) *Tikal: Paleoecology of an Ancient Maya City* (Cambridge University Press, 2015).
